# Real-world treatment patterns and determinants of therapy in systemic sclerosis: findings from the German Network for SSc cohort

**DOI:** 10.1186/s13075-026-03854-2

**Published:** 2026-07-10

**Authors:** Tim Filla, Alexandru Micu, Lasse Hansen, Isabel Grinberg, Franca Sophie Deicher, Laura-Marie Lahu, Alexandru-Emil Matei, Christina Düsing, Gabriela Riemekasten, Norbert Blank, Annette Alberding, Marc Schmalzing, Jan Ehrchen, Claudia Günther, Gabriele Zeidler, Ilona Jandova, Gernot Keyßer, Margitta Worm, Ulf Müller-Ladner, Aaron Juche, Laura Susok, Peter Korsten, Jörg Henes, Alexander Kreuter, Pia Moinzadeh, Thomas Krieg, Marielle Girke, Margarida Alves, Nicolas Hunzelmann, Jörg H.W. Distler, Andrea-Hermina Györfi

**Affiliations:** 1 Department of Rheumatology, University Hospital Düsseldorf, Medical Faculty of Heinrich Heine University, Düsseldorf, Germany; 2 Hiller Research Center, Medical Faculty, University Hospital Düsseldorf, Heinrich Heine University, Düsseldorf, Germany; 3 Fraunhofer Institute for Translational Medicine and Pharmacology ITMP, and Fraunhofer Cluster of Excellence for Immune Mediated Diseases CIMD, Frankfurt am Main, Germany; 4 Clinic for Rheumatology and Clinical Immunology, University Hospital Schleswig-Holstein, University of Lübeck, Lübeck, Germany; 5 Division of Rheumatology, Internal Medicine V, University Hospital Heidelberg, Heidelberg, Germany; 6 Department of Internal Medicine II- Internal Rheumatolgy, St. Josef Hospital Wuppertal, Wuppertal, Germany; 7 Rheumatology/Clinical Immunology, Department of Internal Medicine II, University Hospital Würzburg, Würzburg, Germany; 8 Department of Dermatology, University Hospital Münster, Münster, Germany; 9 Department of Dermatology, University Hospital Carl Gustav Carus, TU Dresden, Dresden, Germany; 10 Department of Rheumatology, Osteology and Pain Therapy, Center for Rheumatology Brandenburg, Johanniter-Hospital Treuenbrietzen, Treuenbrietzen, Germany; 11 Rheumatology and Clinical Immunology, University Medical Center Freiburg, Freiburg, Germany; 12 Department of Internal Medicine, Division of Rheumatology, University Hospital Halle (Saale), Halle, Germany; 13 Department of Dermatology, Venereology and Allergology, Charité – Universitaetsmedizin Berlin, Berlin, Germany; 14 Department of Rheumatology and Clinical Immunology, Justus-Liebig University Giessen, Kerckhoff Clinic, Bad Nauheim, Germany; 15 Department of Rheumatology, Immanuel Hospital Berlin-Buch, Berlin, Germany; 16 Department of Dermatology, Clinic Dortmund, Dortmund, Germany; 17 Department of Rheumatology and Clinical Immunology, St. Josef-Stift Sendenhorst, Sendenhorst, Germany; 18 Centre for Interdisciplinary Rheumatology, Auto-inflammatory Diseases and Internal Medicine 2, University Hospital Tübingen, Tübingen, Germany; 19 Department of Dermatology, Venereology and Allergology, Helios St Elisabeth Hospital Oberhausen, University Witten/Herdecke, Oberhausen, Germany; 20 Department of Dermatology and Venereology, University Hospital Cologne, Cologne, Germany; 21 Translational Matrix Biology, Faculty of Medicine, University of Cologne, Cologne, Germany; 22 Cologne Excellence Cluster on Cellular Stress Responses in Ageing-Associated Diseases (CECAD), University of Cologne, Cologne, Germany; 23 Center for Molecular Medicine (CMMC), University of Cologne, Cologne, Germany; 24 Center of Excellence Real World Evidence, Boehringer Ingelheim Pharma GmbH & Co.KG, Ingelheim am Rhein, Ingelheim am Rhein, Germany; 25 Therapeutic Area Inflammation, Boehringer Ingelheim International GmbH, Ingelheim am Rhein, Ingelheim am Rhein, Germany

**Keywords:** SSc, Treatment, Fibrosis, Inflammation, Vasculopathy, DNSS, Registry

## Abstract

**Objectives:**

Therapeutic management of systemic sclerosis (SSc) has evolved considerably in recent years. However, contemporary treatment patterns and prescribing determinants remain poorly characterized.

**Methods:**

Data from the German Network for SSc (DNSS) cohort containing 6,583 patients were analyzed to describe trends in vasoactive, immunomodulatory, and antifibrotic therapy; assess variation between centers and specialties; delineate co-prescription patterns; and identify clinical predictors of treatment.

**Results:**

Use of endothelin receptor antagonists and prostanoids / prostacyclin receptor agonists increased from 3.0% [95% confidence interval (CI): 1.5%–5.3%] in 2005 to 28.4% [25.5%–31.3%] in 2025, whereas prescription of calcium channel blockers and phosphodiesterase-5 inhibitors remained stable. Immunomodulatory therapy shifted away from cyclophosphamide towards mycophenolate mofetil, rituximab, and nintedanib, accompanied by a marked decline in glucocorticoid use (50.0% [31.9%–68.1%] in 2000 to 18.1% [11.8%–25.9%] in 2025). University or rheumatology centers prescribed immunomodulators and antifibrotics more frequently than non-university or dermatology centers. Co-prescription patterns showed common combination therapy with tocilizumab or rituximab and methotrexate. Nintedanib was commonly co-administered with mycophenolate, but also with cyclophosphamide or methotrexate. Rituximab was most commonly combined with mycophenolate and tocilizumab with methotrexate. Multivariable mixed models identified modified Rodnan skin score, interstitial lung disease, heart involvement, and care in a university, particularly rheumatology, center as major determinants of immunomodulatory and antifibrotic therapy.

**Conclusions:**

SSc treatment has evolved over the past 25 years, with prescribing patterns increasingly reflecting evidence and guideline recommendations. However, differences between specialties and care settings highlight the need for broader implementation of multidisciplinary, guideline-based care.

**Supplementary Information:**

The online version contains supplementary material available at 10.1186/s13075-026-03854-2.

## Key messages

1. The treatment of patients with systemic sclerosis changed considerably within the last years with shifts towards biologics, antifibrotic therapies, and combination therapies.

2. Prescription patterns are directed by the clinical manifestations, particularly interstitial lung disease, pulmonary hypertension, and skin involvement.

3. Substantial treatment variation persists between different specialties and center types (university vs. non-university) in systemic sclerosis care.

**Supplementary Information:**

The online version contains supplementary material available at 10.1186/s13075-026-03854-2.

## Introduction

Management of SSc has undergone substantial transformation over the past two decades, driven by advances in understanding disease pathogenesis, emergence of targeted therapies, and improved recognition of organ-specific complications [1, 2]. Historically, treatment options were limited to symptomatic vasoactive drugs and a limited number of immunosuppressive therapies for severe organ involvement [3]. Over time, accumulating evidence from clinical trials and observational studies has reshaped treatment strategies, particularly with respect to interstitial lung disease (ILD), digital ulcers, and pulmonary arterial hypertension (PAH) [1].

Positive clinical trial results for rituximab (RTX) [4, 5] and tocilizumab (TCZ) [6, 7] have supported their use in clinical practice in SSc thereby expanding the therapeutic armamentarium for inflammatory and fibrotic manifestations. The approval of nintedanib for SSc-associated ILD marked the first direct antifibrotic therapy of SSc [8]. In parallel, vasoactive therapy for SSc has diversified.

The influence of these efficacy data on real-world prescribing is understudied. Prescription patterns may be influenced by medical specialty, institutional experience, and treatment era [9–11]. In Germany, SSc patients are seen in particular by rheumatologists and dermatologists. Dermatologists historically focused on cutaneous and vascular manifestations, whereas rheumatologists more frequently managed organ and musculoskeletal manifestations. Additional differences in management may arise between tertiary centers and other centers managing SSc patients, e.g. university centers may have earlier access to novel targeted agents.

The German Network for Systemic Sclerosis (DNSS) hosts one of the earliest and largest national databases for SSc, with longitudinal data spanning from 2000 to 2025 and more than 6,500 documented patients with more than 24,000 visits. The DNSS database is well positioned to delineate such trends. Its nationwide scope captures variations across institutions, while detailed treatment records allow investigation of drug prescription patterns. Mapping these temporal and structural treatment differences provides critical insight into evolving clinical practice and may identify barriers to equitable care. Moreover, understanding real-world adoption of emerging therapies can guide dissemination strategies and inform clinical guideline development.

This study evaluates two decades of prescription patterns in the DNSS cohort, quantifying changes in immunomodulatory, vasoactive, and antifibrotic therapy over time, and compares these trends across specialties and care settings. It further examines co-prescription clustering. Together, these analyses contribute to defining contemporary treatment paradigms in SSc and highlighting opportunities for further optimization.

## Materials and methods

The data source used for the analysis was the German Network of SSc cohort containing information on 6,583 German SSc patients collected between February 2000 and October 2025 in 25 centers. The cohort contains demographic variables like age, sex, BMI, as well as detailed clinical features including organ involvement and treatment prescription. All variables collected in the DNSS registry are listed in Supplementary Table 1.

### Variable definition

Overall, 20 different therapies were investigated and categorized into vascular, immunomodulatory, and antifibrotic therapies. Vascular therapies included angiotensin-converting enzyme inhibitors (ACEi), angiotensin receptor blockers (ARB), beta-blockers (BB), calcium channel blockers (CCB), endothelin receptor antagonists (ERA), phosphodiesterase-5 inhibitors (PDE5i), prostanoids / prostacyclin receptor agonists, soluble guanylate cyclase (sGC) stimulators, and other vasodilators. Immunomodulatory therapies comprised glucocorticoids (GCs), cyclophosphamide (CYC), chloroquine / hydroxychloroquine, methotrexate (MTX), mycophenolate mofetil / mycophenolic acid (MMF/MFA), azathioprine, RTX, and TCZ. Group-level therapy status for vascular, immunomodulatory, and antifibrotic therapies was defined only when prescription information was available for all therapies within the respective group. If information for any therapy within a group was missing, the group-level status was coded as missing. When complete information was available, the group-level status was coded as **yes** if at least one therapy within the group was prescribed and **no** otherwise.

DNSS centers were divided into rheumatological vs. dermatological centers as well as university vs. non-university centers. To assess differences in time before and after approval of new therapeutic therapies, the year after nintedanib approval in Europe was chosen. While approval was granted in 2020, 2021 was considered more appropriate due to reimbursement processes and delayed implementation in routine care. For therapies such as RTX and TCZ, which are not approved for the use in SSc in Germany, changes after the year of publication of evidence from clinical trials (DESIRES trial in 2021 for RTX [4] and focuSSced trial [6] in 2020 for TCZ) were analyzed.

Any elevated CRP level above 5 mg/l was classified as raised CRP.

### Statistical analysis

Descriptive variable information is provided by using mean ± standard deviation for continuous variable and absolute number with percentage for categorical variables.

To estimate the proportion of patients treated within a specific group the number of patients receiving treatment divided by the number of patients with treatment information was used. Then, exact 95% confidence intervals were calculated using the estimated proportion along with patient number. Proportions across different modalities (e.g., rheumatological vs. dermatological centers) were compared using Fisher’s exact test. All p-values were adjusted using Bonferroni-Holm method and an adjusted p-value below 0.05 was considered statistically significant.

For each calendar year, the proportion of patients receiving a given treatment was calculated using all visits within that year. Treatment status was assigned as yes if at least one visit indicated treatment use. Treatment status was assigned as no if treatment information was available for at least one visit and all available visits indicated no treatment.

To get a more unbiased estimate for modality effect on treatment proportion, a logistic mixed regression model with treatment prescription (yes/no) as the outcome, rheumatological/dermatological center, university center (yes/no) as the independent variable and a set of clinical and demographic variables, including: age, sex, disease duration, ILD, presence of pulmonary hypertension (PH), presence of digital ulcers, modified Rodnan skin score (mRSS), heart involvement, renal involvement, anti-Scl70 and anti-centromere autoantibody positivity, was used. For each model Wald − 95% confidence intervals were calculated.

For all analyses, a complete case analysis was conducted. All statistical analyses were conducted using R version 4.4.3.

## Results

The dataset comprised 6,582 SSc patients with a total of 24,140 clinical visits recorded between January 2000 and October 2025. The mean age of the cohort was 55.3 ± 13.8 years, and the majority were female (*n* = 5,246; 79.6%). Of the total population, 4,527 patients (68.8%) were managed in rheumatological centers, and 2,055 patients (31.2%) were managed in dermatological centers (Supplementary Table 2). Most patients were treated in university centers (*n* = 5,264; 80%), whereas 1,318 patients (20%) received care in non-university centers (Supplementary Table 3). Regarding treatment era, 5,780 patients (87.8%) were treated before the approval of nintedanib in Europe, and 803 patients (12.2%) were treated after its approval (Supplementary Table 4).

### Changes in prescription patterns over time

**Fig. 1:**
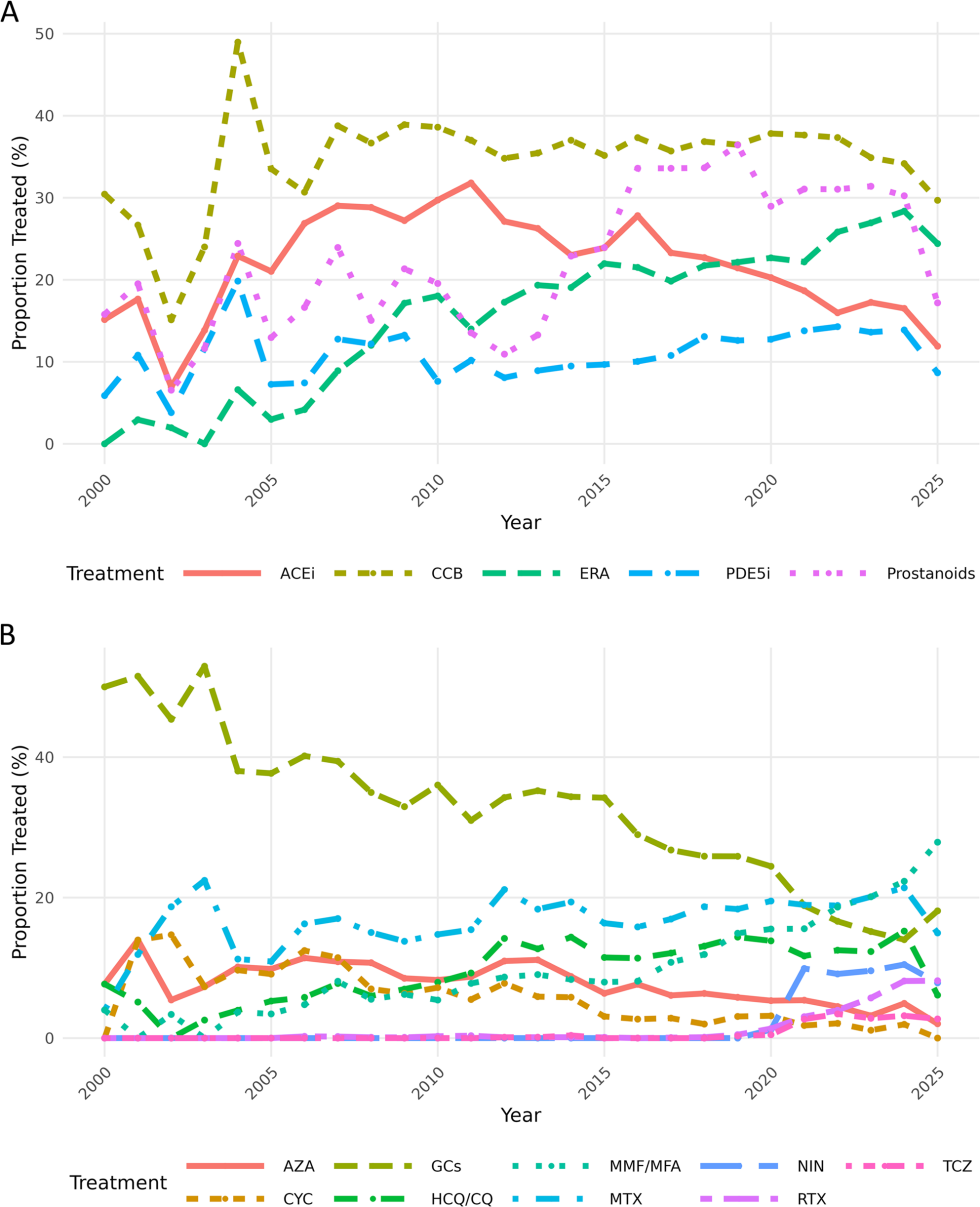
Temporal trends in vascular-targeted, immunomodulatory and antifibrotic medications in SSc between 2000–2025. **A** Annual proportion of patients receiving vasoactive therapies, including ACEi (angiotensin converting enzyme inhibitors), CCBs (calcium channel blockers), ERA (endothelin receptor antagonists), PDE5i (phosphodiesterase type 5 inhibitors), and prostanoids / prostacyclin receptor agonists. **B** Annual proportion of patients treated with immunomodulatory or antifibrotic therapies, including AZA (azathioprine), GCs (glucocorticoids), MMF/MFA (mycophenolate mofetil/mycophenolic acid), CYC (cyclophosphamide), HCQ/CQ (hydroxychloroquine/chloroquine), MTX (methotrexate), RTX (rituximab), TCZ (tocilizumab), and NIN (nintedanib)

We examined temporal changes in treatment prescription over the past 25 years (Fig. 1). For vasoactive therapies, there was a marked increase in ERA prescriptions from 3.0% [1.5%–2.9%] in 2005 to 28.4% [25.5%–31.3%] in 2024. In contrast, use of ACEi declined from 31.8% [29.1%–34.6%] in 2011 to 11.9% [6.8%–18.9%] in 2025. For immunomodulatory therapies, GC prescriptions decreased substantially from 50.0% [31.9%–68.1%] in 2000 to 18.1% [11.8%–25.9%] in 2025. Following their approval or positive results from clinical trials, nintedanib, RTX, and TCZ showed rapid increases in use: all three agents had essentially 0% use prior to approval / publication of primary results of clinical trials (nintedanib in 2020; RTX in 2021, TCZ in 2018) and reached 7.9% [3.8%–14.0%], 8.2% [4.3%–13.8%] and 2.7% [0.7%-6.8%], respectively, by 2025.

Multivariate logistic mixed models revealed as expected, that nintedanib use was strongly independently associated with ILD (OR 39.5 [4.3–365.9], *p* = 0.001). ERA (OR 9.6 [5.9–15], *p* < 0.0001) and PDE5i (OR 14.0 [8.8–22.2], *p* < 0.0001) were predominantly prescribed to patients with PH. Due to the comparatively low number of RTX and TCZ prescriptions, analyses for these therapies were restricted to descriptive comparisons. RTX-treated patients had more frequently dcSSc (48.2% vs. 34.4%), elevated CRP levels (5.6 vs. 3.48), and arthritis (31.4% vs. 21.0%). Similarly, TCZ-treated patients had more frequently dcSSc (57.0% vs. 34.5%), anti-Scl70 autoantibodies (12.1% vs. 6.5%), and shorter disease duration (26.0% vs. 19.6% within the first five years).

**Fig. 2:**
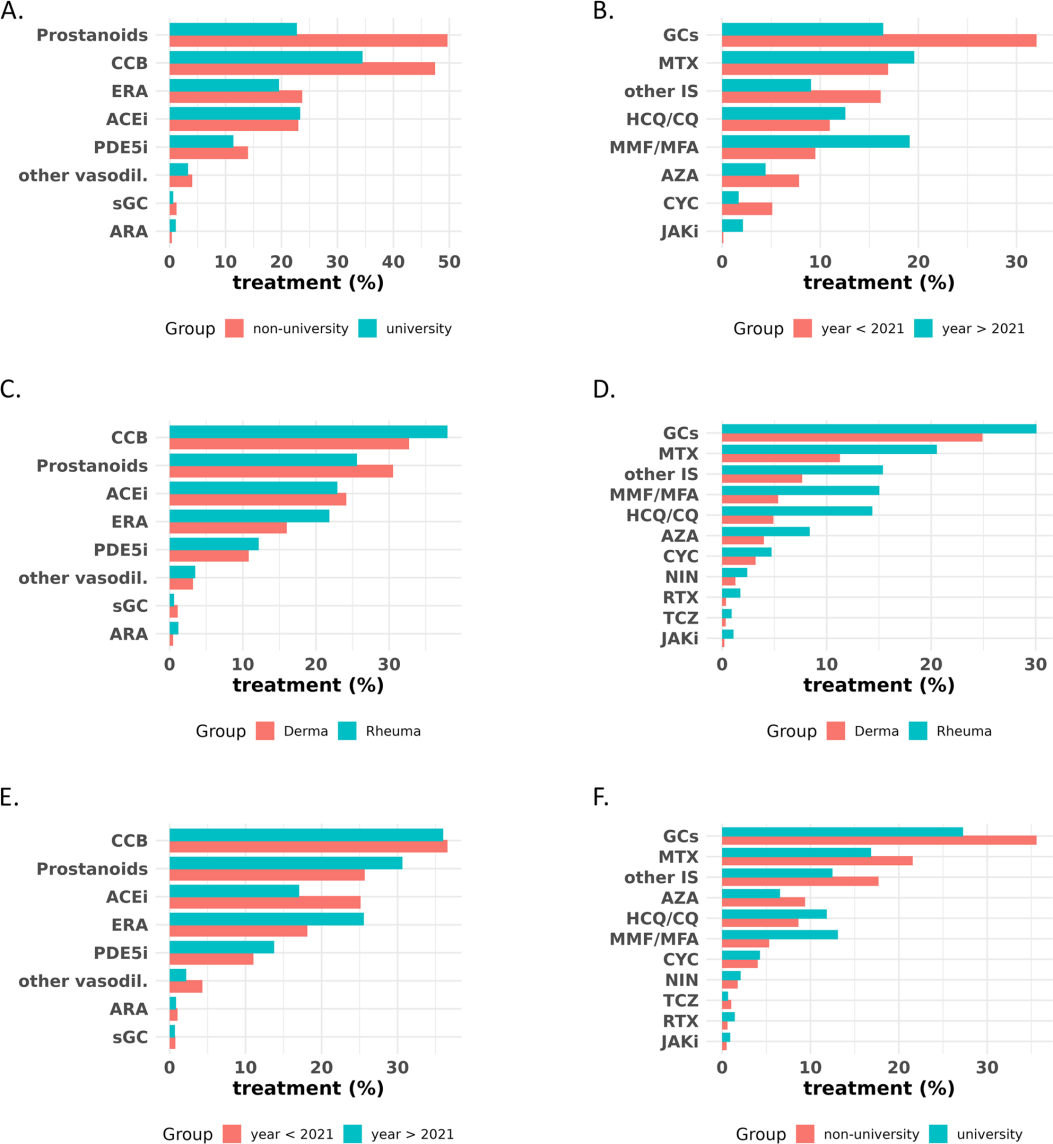
Treatment patterns in German centers by approval period, specialty, and institution type. Comparative bar plots showing proportions of vasoactive (A, C, E) and immunomodulatory therapies (B, D, F) administered in Germany. Figures **A**–**B** compare treatment patterns before (red bars) and after (blue bars) approval of nintedanib in Germany and positive clinical trial results for tocilizumab and rituximab. Figures **C**–D compare dermatological (red bars) and rheumatological centers (blue bars). Figures **E**–**F** compare non-university (red bars) and university centers (blue bars). Abbreviations: Derma., dermatology center; Rheuma., rheumatology center; CCB, calcium channel blockers; ACEi, angiotensin converting enzyme inhibitors; ERA, endothelin receptor antagonists; PDE5i, phosphodiesterase type 5 inhibitors; other vasodil., other vasodilators; sGC, soluble guanylate cyclase stimulators; ARA, angiotensin receptor antagonists;; GCs, glucocorticoids; MTX, methotrexate; Other IS, other immunosuppressives; MMF/MFA, mycophenolate mofetil/mycophenolic acid; HCQ/CQ, hydroxychloroquine/chloroquine; AZA, azathioprine; CYC, cyclophosphamide; NIN, nintedanib; RTX, rituximab; TCZ, tocilizumab; JAKi, Janus kinase inhibitor

In analyses comparing prescription patterns before and after nintedanib approval in Europe and positive clinical trial results for RTX and TCZ in 2021 (Fig. 2A, B), we observed strong differences in immunomodulatory / antifibrotic treatment proportions, while overall vasoactive therapy prescriptions remained comparable. Notable differences were evident at the level of individual agents in particular for GCs (34% before vs. 17% after nintedanib approval, *p* < 0.0001), consistent with its gradually reduced use over time. Interestingly, prescription of MMF / MFA, which may synergize with the effects of nintedanib on SSc-ILD [12], increased from 8% to 21% after 2021, likely also reflecting the dissemination of the favorable results of the 2017 Scleroderma Lung Study II (SLS II).

### Differences in prescription patterns between centers and specialties

Comparative analyses revealed substantial differences in prescription patterns across clinical settings (Fig. 2C, D). Rheumatological centers exhibited numerically higher overall use of immunomodulatory treatments compared with dermatological centers (53% vs. 34%, *p* < 0.0001), and greater nintedanib prescription (3% vs. 1%, *p* < 0.0001). Vasoactive therapy prescription rates were similar between specialties (59% vs. 57%, *p* = 0.22). At the level of individual immunomodulatory agents, the largest specialty differences were observed for MMF (13% vs. 5%, *p* < 0.0001), chloroquine / hydroxychloroquine (13% vs. 6%, *p* < 0.0001), and MTX (20% vs. 12%, *p* < 0.0001).

When comparing non-university versus university centers (Fig. 2E, F), the most pronounced difference was seen for vasoactive therapies, with non-university centers having higher overall prescription rates (65% vs. 57%, *p* < 0.0001). This was in particular due to higher prescription rates of intravenous prostanoids in non-university centers. The overall prescription rate for antifibrotic and immunomodulatory drug use was similar. However, differences were observed for MMF, which was prescribed more frequently in university settings and glucocorticoids, MTX and other immunomodulatory drugs, which were more commonly used in non-university settings.

We also evaluated associations between treatment prescriptions and clinical, demographic, and center-level factors (Supplementary Fig. 1). After adjusting for patient clinical and demographic characteristics, many associations with center type persisted: MMF was more frequently prescribed in rheumatological versus dermatological centers (OR 9.0 [4.1–19.8], *p* < 0.0001) and in university versus non-university centers (OR 4.1 [1.3–12.9], *p* = 0.02). Among vascular therapies, both prostanoids (OR 0.01 [0.01–0.02], *p* < 0.0001) and ERA (OR 0.13 [0.08–0.19], *p* < 0.0001) were prescribed less often in university than in non-university centers.

### Co-prescription patterns

Given the multisystem nature of SSc requiring combination therapies, we assessed co-prescription patterns of medications (Supplementary Figs. 2, 3). For vasoactive agents, ERA co-prescription was high among patients treated with PDE5i (61%) (Supplementary Fig. 2). Among patients receiving immunomodulatory or antifibrotic therapies, nintedanib was most commonly co-prescribed with MMF (46%), followed by CYC (26%), GCs (25%), and MTX (22%). In the RTX subgroup, concomitant therapy frequently included GCs (52%), MMF (44%), or MTX (31%). Among those treated with TCZ, co-prescription with GCs (40%) and MTX (32%) was more common than with MMF (8%) (Supplementary Fig. 3).

## Discussion

In this large, real-world German multicenter cohort spanning more than two decades, we observed marked temporal shifts in the therapeutic management of SSc that closely reflect evolving clinical evidence, regulatory approvals, and guideline recommendations [1, 13, 14]. Our findings complement recent registry data [10, 15, 16], demonstrating practice evolution in SSc across major therapeutic domains.

Consistent with reports from other cohorts [10], we found a substantial increase in vascular therapies over time, particularly ERA use, which rose from 3.0% in 2005 to 28.4% in 2024. This trend aligns with their established roles in severe Raynaud’s phenomenon, digital ulcer prevention, and SSc-PAH, as well as guideline recommendations emphasizing early screening and combination vasodilator therapy for SSc-PAH [1]. Although primarily prescribed for severe Raynaud’s phenomenon / ischemic fingertip ulcers, the increasing use of combined vasodilator therapies (e.g., ERA and PDE5i) reflects contemporary PAH management algorithms based on PAH-specific RCTs and registry studies demonstrating improved outcomes with early combination treatment [17].

We further noted a decline in GC prescribing, from 50.0% in 2000 to 18.1% in 2025, supporting previous EUSTAR reports and reflecting growing recognition of limited efficacy and the potential risk of scleroderma renal crisis, particularly with high-dose GCs [18]. All European, German and British guidelines caution against routine high-dose GC use, reserving GCs for specific indications due to adverse risk profiles [1, 14, 19]. However, despite decreased use, GCs are still used in 18.1% of SSc patients.

However, a limitation of our study is the incomplete documentation of the exact doses of GCs used in the DNSS database. Thus, future studies are required to evaluate whether not only the overall use, but also the doses of GC decreased over time.

The adoption of novel therapies in recent years was rapid. The integration of nintedanib for the management of SSc-ILD, reaching a use of up to 7.9% by 2025, reflects RCT-based evidence from the SENSCIS trial [8], which demonstrated a clinically relevant reduction in the rate of FVC decline, and its endorsement in the 2023 EULAR/EUSTAR treatment recommendations and the 2025 ERS/EULAR guidelines for the management of CTD-ILD [1, 13, 20]. Similarly, increasing use of RTX, of up to 8.2% by 2025, parallels expanding RCT and observational evidence for its efficacy in skin and lung fibrosis, and its conditional support in updated guidelines [4, 5]. Although more modestly used in our cohort (2.7% by 2025), the role of TCZ is consistent with its guideline-recommended use in selected SSc patients with early dcSSc and ILD, based on RCTs showing stabilization of ILD and trends toward improved skin outcomes in this subset of patients [6, 7].

Our data also revealed major variations in prescriptions across specialties and care settings. Rheumatological centers had greater use of immunomodulatory therapies and novel agents such as nintedanib compared with dermatological centers, likely reflecting differences in organ-based expertise, familiarity with systemic manifestations, and access to advanced therapies. Likewise, observed differences in vasoactive therapy utilization between university and non-university centers may be attributable to variations in referral patterns, subspecialty expertise, and healthcare structures, with non-university centers often providing more locally accessible inpatient vasodilator treatments and managing a higher proportion of patients with Raynaud’s phenomenon or digital ulcers rather than severe SSc-PAH. Such heterogeneity in practice patterns underscores the need for broader dissemination and implementation of consensus recommendations, as well as the need for multidisciplinary management of SSc patients.

The observed co-prescription patterns illustrate the multidimensional therapeutic strategies required in SSc. Frequent co-use of ERA with PDE5i and nintedanib with immunomodulators like MMF reflects guideline emphasis on individualized, organ-based treatment and emerging evidence supporting combination therapy, particularly for SSc-ILD. Notably, recent guideline updates suggest that nintedanib together with MMF may be considered in SSc-ILD, albeit with conditional evidence [1, 12, 20].

This study has limitations inherent to the analysis of an observational cohort, including inability to establish causality and potential influence of unmeasured confounders. Changes in classification criteria, referral practices, and data collection over time may also contribute to observed trends.

In summary, this study shows that the treatment of SSc has changed markedly over the past 25 years, with prescribing patterns aligning with recent evidence from clinical trials and updated guideline recommendations. The reduced use of glucocorticoids, alongside greater adoption of vascular therapies, nintedanib, and biologic immunomodulators, suggests that recent evidence is being incorporated into routine clinical practice. At the same time, substantial differences in treatment approaches between specialties and between expert and non-expert centers remain evident. These findings highlight the need for dissemination of guideline-based care and intensified multidisciplinary collaboration to ensure that patients with SSc receive comparable and evidence-based management across care settings.

## Supplementary Information

Supplementary Material 1.

## Data Availability

All data from this study are presented in the manuscript and figures. Raw data are available from the corresponding author upon reasonable request.

## Abbreviations

ACA: Anti-centromere antibodies
ACEi: Converting enzyme inhibitors
ANA: Antinuclear antibodies
ARA: Angiotensin receptor antagonists
ARB: Angiotensin receptor blockers
AZA: Azathioprine
BB: Beta-blockers
CCB: Calcium channel blockers
CK: Creatin kinase
CO: Cardiac output
CRP: C reactive protein
CTD: Connective tissue disease
CYC: Cyclophosphamide
dcSSc: Diffuse cutaneous systemic sclerosis
Derma.: Dermatology center
diaPAP: Diastolic pulmonary artery pressure
DLCOc/SB: Corrected diffusing capacity of the lung for carbon oxide / single breath
DNSS: German Network for Systemic Sclerosis
eGFR: Estimated glomerular filtration rate
ERA: Endothelin receptor antagonists
ERS: European Respiratory Society
ESR: Erythrocyte sedimentation rate
EULAR: European Alliance of Associations for Rheumatology
EUSTAR: European Scleroderma Trials and Research Group
FEV1: Forced expiratory volume in one seconds
FVC: Forced vital capacity
GAVE: Gastric antral vascular ectasia
Hb: Hemoglobin
HCQ/CQ: Hydroxychloroquine/chloroquine
ILD: Interstitial lung disease
IS: Immunosuppressives
JAKi: Janus Kinase Inhibitors
lcSSc: Limited cutaneous systemic sclerosis
LV-EF: Left ventricular ejection fracture
MMF/MFA: Mycophenolate mofetil / mycophenolic acid
mRSS: Modified Rodnan skin score
MTX: Methotrexate
NIN: Nintedanib
NT-proBNP: N terminal pro-brain natriuretic peptide
NYHA: New York Heart Association
PAH: Pulmonary arterial hypertension
PCWP: Pulmonary capillary wedge pressure
PDE5i: Phosphodiesterase-5 inhibitors
PH: Pulmonary hypertension
PVR: Pulmonary vascular resistance
RCT: Randomized clinical trial
Rheuma: Rheumatology center
RTX: Rituximab
TCZ: Tocilizumab
SCT: Stem cell therapy
sGC: Soluble guanylate cyclase
SSc: Systemic sclerosis
sysPAP: Systolic pulmonary artery pressure
TnT: Troponin T
UCTD: Undifferentiated connective tissue disease

## Declarations

This was a collaborative research study where Boehringer Ingelheim was involved in the design, analysis or interpretation of the results but was not the regulatory sponsor.

Boehringer Ingelheim was given the opportunity to review the manuscript for medical and scientific accuracy as it relates to Boehringer Ingelheim substances, as well as intellectual property considerations.

CD received honoraria for lecturing, consulting and travel expenses for attending meetings from Novartis, GSK, Medac, AstraZeneca, AlfaSigma, Johnson&Johnson, Sanofi, Lilly, Amgen, AbbVie, Kyverna, Pfizer and Deutsche Rheuma-Liga.

PK has received honoraria or travel support from Abbvie, Alfasigma, Amgen, AstraZeneca, Boehringer Ingelheim, Bristol-Myers-Squibb, Chugai, GlaxoSmithKline, Janssen-Cilag, Lilly, Novartis, Pfizer, and Sanofi, all unrelated to this paper.

MG and MA are employees of Boehringer-Ingelheim.

P. M. reports lecture fees received from Boehringer Ingelheim Pharma GmbH & Co., Janssen Cilag GmbH, ECM GmbH, AEMH e.V, Leo Pharma GmbH and adboard fees received from Almirall Hermal GmbH, Boehringer Ingelheim Pharma GmbH & Co.

JHWD has received grants or holds contracts from: AbbVie, Anamar, Argenx, ARXX, BMS, Bayer Pharma, Boehringer Ingelheim, Cantargia, Celgene, CSL Behring, ExoTherapeutics, Galapagos, GSK, Inventiva, Kiniksa, Lassen, Novartis, Sanofi-Aventis, RedX and UCB; consulting fees from: AbbVie, Active Biotech, Anamar, ARXX, AstraZeneca, Bayer Pharma, Boehringer Ingelheim, Celgene, Galapagos, Genentech, GSK, Inventiva, Janssen, Novartis, Pfizer, Roche and UCB; Payment or honoraria for lectures, presentations, speakers bureaus, manuscript writing or educational events from: AbbVie, Active Biotech, Anamar, ARXX, AstraZeneca, Bayer Pharma, Boehringer Ingelheim, Celgene, Galapagos, Genentech, GSK, Inventiva, Janssen, Novartis, Pfizer, Roche and UCB; Payment for expert testimony from: AbbVie, Anamar, ARXX, AstraZeneca, Bayer Pharma, Boehringer Ingelheim, Cantargia, Celgene, Galapagos, Genentech, GSK, Inventiva, Janssen, Novartis, Pfizer, Roche and UCB; Support for attending meetings and/or travel from: AbbVie, SOBI; Stock or stock options from: 4D Science; FibroCure; Receipt of equipment, materials, drugs, medical writing, gifts or other services from: Boehringer Ingelheim.

AHG has received honoraria for presentations from Boehringer Ingelheim and AbbVie.
