# Supplementary material for: Real-world treatment patterns and determinants of therapy in systemic sclerosis: findings from the German Network for SSc cohort
Source: Arthritis Res Ther. 2026 Jul 10;28:140. doi: 10.1186/s13075-026-03854-2 (PMC13355332; doi:10.1186/s13075-026-03854-2)
Supplement: Supplementary file 1 — Supplementary Material 1. [file 13075_2026_3854_MOESM1_ESM.docx]

**Supplementary Figures:**


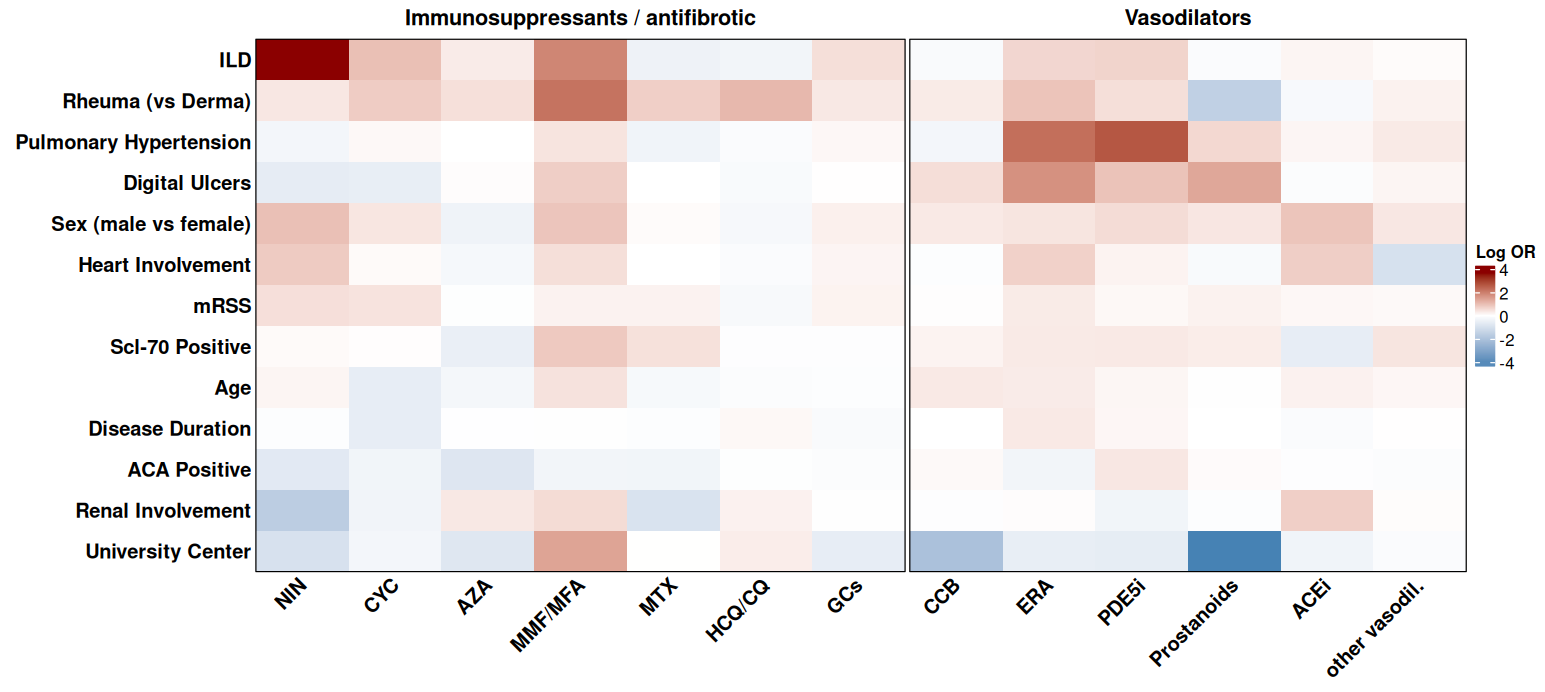


**Supplementary Figure 1.: Associations between clinical characteristics and odds of receiving specific treatments.** Heatmap displaying the results of a multivariable logistic mixed model providing log odds ratios (Log OR) for the association between key demographic, clinical, serologic, and organ-involvement features and the use of individual treatments. Positive values (red) indicate higher odds of receiving the treatment, whereas negative values (blue) indicate lower odds. Notable associations include: pulmonary hypertension strongly predicting ERA and prostanoid use; ILD predicting immunosuppressive and antifibrotic therapy, including MMF and CYC, and NIN, respectively; and university-center care associated with more frequent use of MMF.

*Abbreviations:* IS, immunosuppressants; ILD, interstitial lung disease; Rheuma., rheumatology center; Derma., dermatology center; mRSS, modified Rodnan skin score; ACA, anti-centromere antibodies; NIN, nintedanib; other vasodil., other vasodilators; CYC, cyclophosphamide; AZA, azathioprine; MMF/MFA, mycophenolate mofetil/mycophenolic acid; MTX, methotrexate; HCQ/CQ, hydroxychloroquine/chloroquine; GCs, glucocorticoids; CCB, calcium channel blockers; ERA, endothelin receptor antagonists; PDE5i, phosphodiesterase type 5 inhibitors; ACEi, angiotensin converting enzyme inhibitors.

**
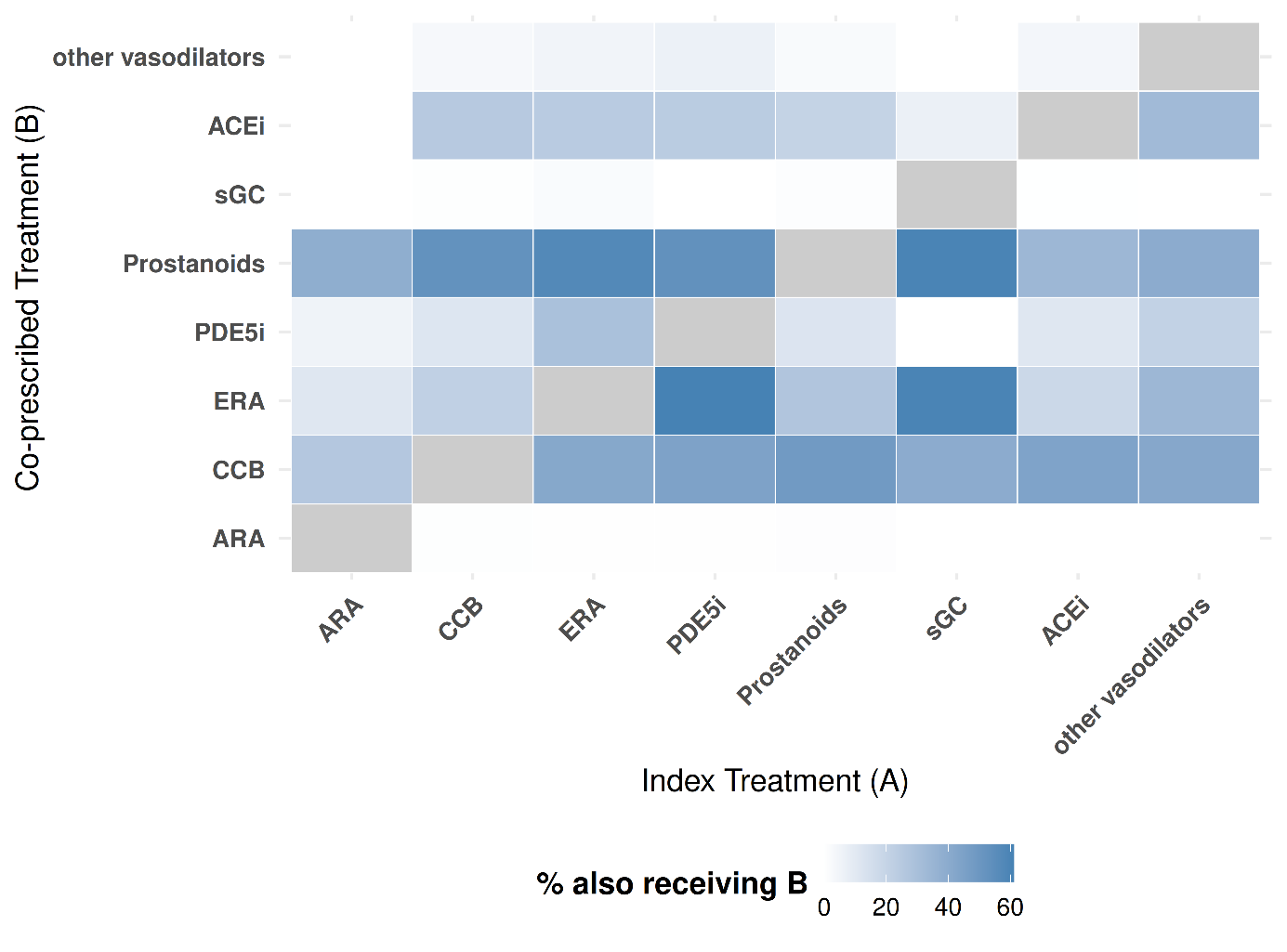
**

**Supplementary Figure 2.** **Co-prescription patterns among vasoactive treatments in the DNSS cohort.** Heatmap showing the percentage of patients receiving treatment B concurrently with treatment A.

*Abbreviations:* ARA, angiotensin receptor antagonists; CCB, calcium channel blockers; ERA, endothelin receptor antagonists; PDE5i, phosphodiesterase type 5 inhibitors; ACEi, angiotensin converting enzyme inhibitors.

**
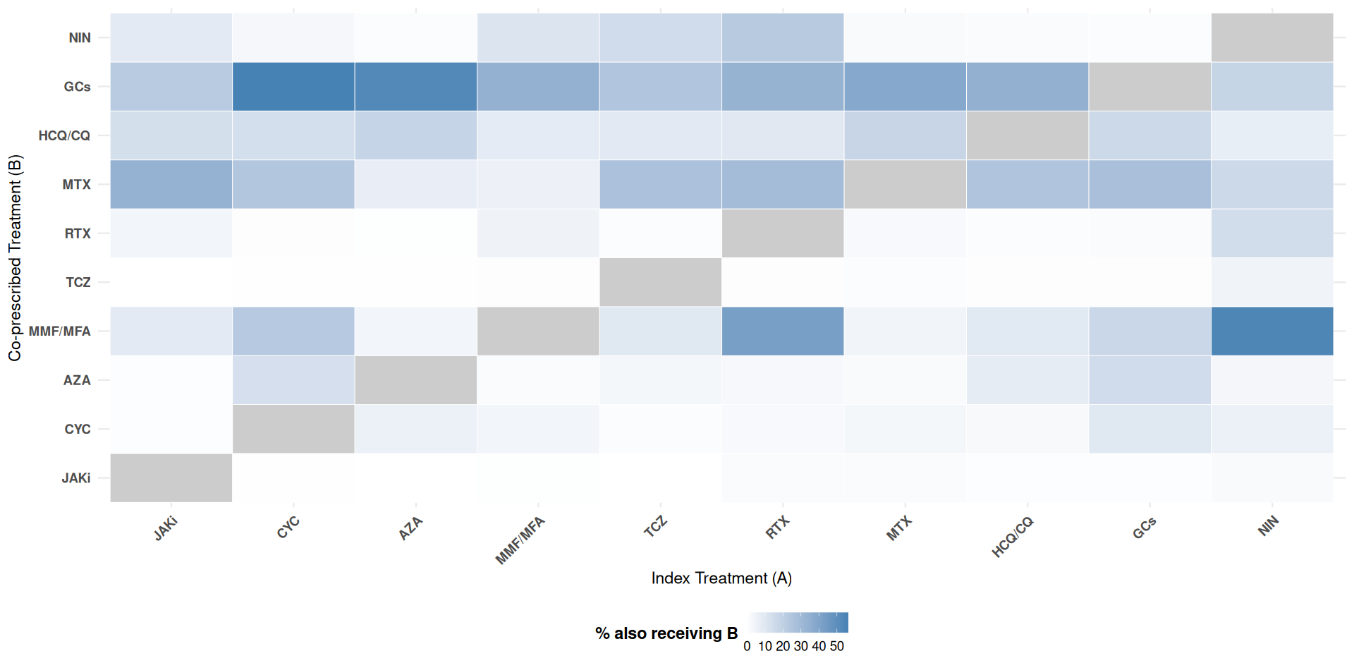
**

**Supplementary Figure 3. Co-prescription patterns among immunomodulatory and antifibrotic treatments in the DNSS cohort.**

*Abbreviations:* JAKi, Janus kinase inhibitors; CYC, cyclophosphamide; AZA, azathioprine; MMF/MFA, mycophenolate mofetil/mycophenolic acid; TCZ, tocilizumab; RTX, rituximab; MTX, methotrexate; HCQ/CQ, hydroxychloroquine/chloroquine; GCs, glucocorticoids; NIN, nintedanib.

**Supplementary Table 1. Parameters collected in the DNSS registry**

| Category | Variable |
| --- | --- |
| Patient demographics | Age in years |
|  | Sex |
| Disease subset & classification | SSc subset (lcSSc, dcSSc, sine scleroderma, overlap syndrome, UCTD) |
| Organ involvement | Skin thickening |
|  | Raynaud’s phenomenon |
|  | Digital ulcers / Pitting scars |
|  | Calcinosis |
|  | Sicca symptoms |
|  | Pulmonary fibrosis |
|  | Pulmonary hypertension (PAH, PH due to left heart disease, PH due to lung disease) |
|  | Cardiac involvement |
|  | Renal involvement |
|  | Musculoskeletal involvement |
| Capillaroscopic changes | Normal / pathologic (hemorrhages, megacapillaries, avascular regions) |
| Autoantibody profile | ANA, anti-Scl70, anti-RNAPIII, anti-CENP, others |
| Laboratory parameters | ESR, CRP, Hb, eGFR, creatinine, proteinuria, uric acid, NT-proBNP, TnT, CK |
| Current manifestations |  |
| - Vascular | Telangiectasia |
|  | Fingertip ulcers / pitting scars / necrosis / ulcers with other localizations |
| - Musculoskelettal | Puffy fingers / arthritis / joint contractures / tendon friction rubs |
| - Gastrointestinal | Dysphagia, reflux, GAVE, malabsorption, diarrhea, stool incontinence |
| - Renal | Scleroderma renal crisis, dialysis |
| - Pulmonary | Dyspnea (NYHA), oxygen therapy |
|  | Lung function: FVC %, DLCOc/SB %, FEV1 % |
| - Cardiac | Cardiovascular events, coronary heart disease, pacemaker implantation, arterial hypertension |
|  | Echocardiography: sysPAP, LV-EF, diastolic dysfunction |
|  | Right heart catheter: mean / sys / diaPAP, PVR, PCWP, CO |
| - Cutaneous | mRSS |
| Therapy |  |
| - Vasoactive | ACEi, ARBs, BBs, CCBs, ERAs, PDE5i, prostanoids, selexipag, riociguat, others |
| - Anticoagulants |  |
| - Antiplatelet drugs |  |
| - Immunosuppressives | GCs, cyclophosphamide, methotrexate, azathioprine, mycophenolate mofetil, chloroquine / hydroxychloroquine, rituximab, tocilizumab, JAKi |
| - Antifibrotics | Nintedanib |
| - Others | SCT, other organ transplantation, analgesics, PPIs, prokinetics, others |
| Comorbidity / Other | Malignancy |
|  | Smoking status |
| Death / Loss of follow-up |  |

*Abbreviations:* lcSSc, limited cutaneous systemic sclerosis; dcSSc, diffuse cutaneous systemic sclerosis; UCTD, undifferentiated connective tissue disease; PAH, pulmonary arterial hypertension; PH, pulmonary hypertension; ANA, antinuclear antibodies; Anti-RNAPIII, anti-RNA-polymerase III antibodies; anti-CENP, anti-centromere antibodies; ESR, erythrocyte sedimentation rate; CRP, C reactive protein; Hb, hemoglobin; eGFR, estimated glomerular filtration rate; NT-proBNP, N terminal pro-brain natriuretic peptide; TnT, troponin T; CK, creatin kinase; GAVE, gastric antral vascular ectasia; NYHA, New York Heart Association; FVC, forced vital capacity; DLCOc/SB, corrected diffusing capacity of the lung for carbon oxide / single breath; FEV1, forced expiratory volume in one seconds; sysPAP, systolic pulmonary artery pressure; LV-EF, left ventricular ejection fracture; diaPAP, diastolic pulmonary artery pressure; PVR, pulmonary vascular resistance; PCWP, pulmonary capillary wedge pressure; CO, cardiac output; mRSS, modified Rodnan skin score; ACEi, angiotensin converting enzyme inhibitors; ARBs, angiotensin receptor blockers; BBs, betablockers; CCBs, calcium channel blockers; ERAs, endothelin receptor antagonists; PDE5i, phosphodiesterase 5 inhibitors; GCs, glucocorticoids; JAKi, Janus kinase inhibitors; SCT, stem cell therapy; PPIs, proton pump inhibitors.

**Supplementary Table 2. Baseline demographic and clinical characteristics of SSc patients treated in dermatological vs rheumatological centers**

| Variable | Overall | Derma | Rheuma |
| --- | --- | --- | --- |
| N | 6582 | 2055 | 4527 |
| Male (%) | 1336.0 (20.4) | 406.0 (20.0) | 930.0 (20.6) |
| Age (mean (SD)) | 55.28 (13.79) | 56.38 (13.75) | 54.78 (13.78) |
| Follow-up (years, mean (SD)) | 4.10 (5.02) | 4.87 (5.54) | 3.74 (4.73) |
| Number of visits (mean (SD)) | 3.67 (3.48) | 3.68 (3.48) | 3.66 (3.48) |
| Disease duration (%) |  |  |  |
| - ≤ 3 years | 3038.0 (49.3) | 860.0 (44.2) | 2178.0 (51.6) |
| - 4-6 years | 971.0 (15.7) | 295.0 (15.2) | 676.0 (16.0) |
| - 7-10 years | 854.0 (13.9) | 301.0 (15.5) | 553.0 (13.1) |
| - ≥ 10 years | 1303.0 (21.1) | 488.0 (25.1) | 815.0 (19.3) |
| ANA positive (%) | 5668.0 (91.0) | 1727.0 (91.0) | 3941.0 (91.1) |
| Anti-Scl70 positive (%) | 1889.0 (32.4) | 552.0 (30.4) | 1337.0 (33.3) |
| ACA positive (%) | 2263.0 (39.0) | 730.0 (40.9) | 1533.0 (38.2) |
| Anti-RNAPIII positive (%) | 275.0 (4.8) | 66.0 (3.4) | 209.0 (5.5) |
| mRSS (mean (SD)) | 8.69 (8.52) | 9.60 (8.67) | 8.29 (8.42) |
| Organ involvement |  |  |  |
| Skin involvement (%) | 5041.0 (77.8) | 1593.0 (78.7) | 3448.0 (77.4) |
| mRSS (mean (SD)) | 8.69 (8.52) | 9.60 (8.67) | 8.29 (8.42) |
| Raynaud phenomenon (%) | 6141.0 (94.7) | 1930.0 (95.2) | 4211.0 (94.4) |
| Digital ulcers (%) | 1375.0 (30.8) | 370.0 (28.2) | 1005.0 (31.8) |
| Calcinosis (%) | 651.0 (14.6) | 220.0 (16.7) | 431.0 (13.6) |
| Telangiectasia (%) | 1482.0 (52.2) | **393.0 (60.6)** | **1089.0 (49.7)** |
| Pulmonary involvement |  |  |  |
| - PH (%) | 747.0 (11.5) | 234.0 (11.6) | 513.0 (11.5) |
| - PAH (%) | 338.0 (5.2) | 114.0 (5.6) | 224.0 (5.0) |
| - ILD (%) | 2095.0 (32.4) | 644.0 (31.8) | 1451.0 (32.6) |
| - - ILD with PH (%) | 410.0 (6.3) | 122.0 (6.0) | 288.0 (6.5) |
| - - ILD without PH (%) | 1731.0 (26.8) | 540.0 (26.7) | 1191.0 (26.8) |
| Baseline FVC % (mean (SD)) | 88.21 (20.00) | 88.57 (19.48) | 88.09 (20.17) |
| Baseline DLCO % (mean (SD)) | 68.31 (21.74) | 69.88 (23.33) | 67.59 (20.93) |
| Cardiac involvement (%) | 732.0 (11.3) | 222.0 (11.0) | 510.0 (11.5) |
| Musculoskeletal involvement (%) | 1981.0 (31.4) | **477.0 (23.8)** | **1504.0 (34.8)** |
| Esophageal involvement (%) | 2999.0 (46.3) | 992.0 (48.9) | 2007.0 (45.1) |
| Stomach involvement (%) | 782.0 (12.1) | 191.0 (9.4) | 591.0 (13.3) |
| Diarrhea (%) | 1977.0 (31.5) | 616.0 (32.0) | 1361.0 (31.2) |
| Malabsorption (%) | 914.0 (22.7) | 239.0 (21.7) | 675.0 (23.0) |
| Renal involvement (%) | 455.0 (7.0) | 163.0 (8.1) | 292.0 (6.6) |
| Scleroderma renal crisis (%) | 51.0 (1.2) | 14.0 (1.1) | 37.0 (1.2) |
| Therapy |  |  |  |
| Vasoactive therapy in the last year (%) | 4095.0 (65.2) | 1261.0 (66.5) | 2834.0 (64.7) |
| ERA (%) | 652.0 (13.9) | 154.0 (11.8) | 498.0 (14.7) |
| PDE5i (%) | 392.0 (8.3) | 105.0 (8.0) | 287.0 (8.5) |
| Prostanoids / prostacyclin receptor agonists (%) | 1488.0 (30.1) | **494.0 (35.0)** | **994.0 (28.1)** |
| sGC stimulators (%) | 15.0 (0.5) | 5.0 (0.8) | 10.0 (0.5) |
| Nintedanib (%) | 68.0 (1.1) | 7.0 (0.4) | 61.0 (1.4) |
| Immunomodulatory therapy last year (%) | 2598.0 (100.0) | 538.0 (100.0) | 2060.0 (100.0) |
| MMF / MFA (%) | 398.0 (7.6) | **69.0 (4.3)** | **329.0 (9.1)** |
| CYC (%) | 400.0 (7.4) | **81.0 (4.9)** | **319.0 (8.6)** |
| AZA (%) | 397.0 (7.4) | 84.0 (5.1) | 313.0 (8.4) |
| MTX (%) | 999.0 (18.0) | **215.0 (12.5)** | **784.0 (20.5)** |
| TCZ (%) | 25.0 (0.4) | 2.0 (0.1) | 23.0 (0.5) |
| RTX (%) | 29.0 (0.4) | 2.0 (0.1) | 27.0 (0.6) |
| Chloroquine / Hydroxychloroquine (%) | 513.0 (9.7) | **89.0 (5.4)** | **424.0 (11.6)** |
| GCs (%) | 1995.0 (32.2) | **491.0 (26.3)** | **1504.0 (34.7)** |
| Other immunomodulatory therapy (%) | 221.0 (10.5) | 35.0 (6.9) | 186.0 (11.6) |
| JAKi (%) | 4.0 (0.1) | 0.0 (0.0) | 4.0 (0.2) |
| ARA (%) | 19.0 (0.5) | 6.0 (0.5) | 13.0 (0.4) |
| BB (%) | 10.0 (0.2) | 0.0 (0.0) | 10.0 (0.3) |
| CCB (%) | 1837.0 (34.0) | 480.0 (30.9) | 1357.0 (35.2) |
| ACEi (%) | 1281.0 (20.8) | 363.0 (19.4) | 918.0 (21.3) |
| Other vasodilators (%) | 56.0 (2.7) | 8.0 (1.6) | 48.0 (3.1) |
| Malignancy ever (%) | 643.0 (16.4) | 180.0 (16.7) | 463.0 (16.3) |

*Abbreviations:* ANA, antinuclear antibodies; ACA, anti-centromere antibodies; Anti-RNAPIII, anti-RNA-polymerase III antibodies; mRSS, modified Rodnan skin score; PH, pulmonary hypertension; PAH, pulmonary arterial hypertension; ILD, interstitial lung disease; FVC, forced vital capacity; DLCO, diffusing capacity of the lung for carbon oxide / single breath; ERAs, endothelin receptor antagonists; PDE5i, phosphodiesterase 5 inhibitors; sGC, soluble guanylate cyclase; MMF / MFA, mycophenolate mofetil / mycophenolic acid; CYC, cyclophosphamide; AZA, azathioprine; MTX, methotrexate; TCZ, tocilizumab; RTX, rituximab; GCs, glucocorticoids; JAKi, Janus kinase inhibitors; ARA, angiotensin receptor antagonists; BBs, betablockers; CCBs, calcium channel blockers; ACEi, angiotensin converting enzyme inhibitors.

**Supplementary Table 3. Baseline demographic and clinical characteristics of SSc patients treated in non-university vs university centers**

| Variable | Overall | Non-  university center | University center |
| --- | --- | --- | --- |
| N | 6582 | 1318 | 5264 |
| Male sex (%) | 1336.0 (20.4) | 288.0 (21.9) | 1048.0 (20.0) |
| Age (mean (SD)) | 55.28 (13.79) | 56.37 (13.41) | 55.00 (13.87) |
| Follow-up (years, mean (SD)) | 4.10 (5.02) | 2.70 (3.89) | 4.44 (5.21) |
| Number of visits (mean (SD)) | 3.67 (3.48) | 2.76 (2.76) | 3.90 (3.61) |
| Disease duration (%) |  |  |  |
| - ≤ 3 years | 3038.0 (49.3) | 544.0 (46.8) | 2494.0 (49.8) |
| - 4-6 years | 971.0 (15.7) | 201.0 (17.3) | 770.0 (15.4) |
| - 7-10 years | 854.0 (13.9) | 189.0 (16.3) | 665.0 (13.3) |
| - ≥ 10 years | 1303.0 (21.1) | 228.0 (19.6) | 1075.0 (21.5) |
| ANA positive (%) | 5665.0 (91.0) | 1144.0 (91.7) | 4521.0 (90.9) |
| Anti-Scl70 positive (%) | 1887.0 (32.4) | 360.0 (32.8) | 1527.0 (32.3) |
| ACA positive (%) | 2261.0 (39.0) | 400.0 (39.0) | 1861.0 (39.0) |
| Anti-RNAPIII positive (%) | 191.0 (3.6) | 11.0 (1.2) | 180.0 (4.0) |
| Organ involvement |  |  |  |
| Skin involvement (%) | 5041.0 (77.8) | 916.0 (70.9) | 4125.0 (79.6) |
| mRSS (mean (SD)) | 8.69 (8.52) | 8.54 (8.16) | 8.73 (8.61) |
| Raynaud phenomenon (%) | 6141.0 (94.7) | 1247.0 (96.4) | 4894.0 (94.2) |
| Digital ulcers (%) | 1375.0 (30.8) | **215.0 (24.5)** | **1160.0 (32.3)** |
| Calcinosis (%) | 651.0 (14.6) | 108.0 (12.3) | 543.0 (15.1) |
| Telangiectasia (%) | 1482.0 (52.2) | 344.0 (53.3) | 1138.0 (51.8) |
| Pulmonary involvement |  |  |  |
| - PH (%) | 747.0 (11.5) | 152.0 (11.8) | 595.0 (11.5) |
| - PAH (%) | 338.0 (5.2) | 63.0 (4.9) | 275.0 (5.3) |
| - ILD (%) | 2095.0 (32.4) | 395.0 (30.7) | 1700.0 (32.8) |
| - - ILD with PH (%) | 410.0 (6.3) | 88.0 (6.9) | 322.0 (6.2) |
| - - ILD without PH (%) | 1731.0 (26.8) | 318.0 (24.8) | 1413.0 (27.3) |
| Baseline FVC % (mean (SD)) | 88.21 (20.00) | 87.93 (19.42) | 88.28 (20.15) |
| Baseline DLCO % (mean (SD)) | 68.31 (21.74) | 66.78 (20.89) | 68.65 (21.91) |
| Cardiac involvement (%) | 732.0 (11.3) | **105.0 (8.1)** | **627.0 (12.1)** |
| Musculoskeletal involvement (%) | 1981.0 (31.4) | **306.0 (26.1)** | **1675.0 (32.6)** |
| Esophageal involvement (%) | 2999.0 (46.3) | 553.0 (42.9) | 2446.0 (47.2) |
| Stomach involvement (%) | 782.0 (12.1) | 225.0 (17.5) | 557.0 (10.7) |
| Malabsorption (%) | 914.0 (22.7) | **130.0 (16.3)** | **784.0 (24.2)** |
| Diarrhea (%) | 1977.0 (31.5) | **294.0 (24.8)** | **1683.0 (33.1)** |
| Renal involvement (%) | 455.0 (7.0) | 113.0 (8.8) | 342.0 (6.6) |
| Scleroderma renal crisis (%) | 51.0 (1.2) | 3.0 (0.4) | 48.0 (1.4) |
| Therapy |  |  |  |
| Vasoactive therapy in the last year (%) | 4095.0 (65.2) | 908.0 (72.4) | 3187.0 (63.4) |
| ERA (%) | 652.0 (13.9) | 138.0 (14.0) | 514.0 (13.8) |
| PDE5i (%) | 392.0 (8.3) | 67.0 (7.0) | 325.0 (8.7) |
| Prostanoids / prostacyclin receptor agonists (%) | 1488.0 (30.1) | **530.0 (49.8)** | **958.0 (24.7)** |
| sGC (%) | 15.0 (0.5) | 4.0 (0.6) | 11.0 (0.5) |
| Nintedanib (%) | 68.0 (1.1) | 17.0 (1.4) | 51.0 (1.0) |
| Immunomodulatory therapy last year (%) | 2598.0 (100.0) | 531.0 (100.0) | 2067.0 (100.0) |
| MMF / MFA (%) | 398.0 (7.6) | **41.0 (4.0)** | **357.0 (8.5)** |
| CYC (%) | 400.0 (7.4) | 74.0 (7.0) | 326.0 (7.5) |
| AZA (%) | 397.0 (7.4) | **114.0 (10.5)** | **283.0 (6.6)** |
| MTX (%) | 999.0 (18.0) | **230.0 (20.8)** | **769.0 (17.4)** |
| TCZ (%) | 25.0 (0.4) | 4.0 (0.3) | 21.0 (0.4) |
| RTX (%) | 29.0 (0.4) | 3.0 (0.2) | 26.0 (0.5) |
| Chloroquine / Hydroxychloroquine (%) | 513.0 (9.7) | 86.0 (8.2) | 427.0 (10.0) |
| GCs stimulators (%) | 1995.0 (32.2) | **463.0 (37.9)** | **1532.0 (30.8)** |
| Other immunomodulatory therapy (%) | 221.0 (10.5) | 41.0 (7.6) | 180.0 (11.5) |
| JAKi (%) | 4.0 (0.1) | 0.0 (0.0) | 4.0 (0.2) |
| ARA (%) | 19.0 (0.5) | 3.0 (0.3) | 16.0 (0.5) |
| BB (%) | 10.0 (0.2) | 2.0 (0.2) | 8.0 (0.2) |
| CCB (%) | 1837.0 (34.0) | **459.0 (41.8)** | **1378.0 (32.0)** |
| ACEi (%) | 1281.0 (20.8) | 270.0 (22.1) | 1011.0 (20.4) |
| Other vasodilators (%) | 56.0 (2.7) | 8.0 (1.5) | 48.0 (3.1) |
| Malignancy ever (%) | 643.0 (16.4) | 107.0 (13.9) | 536.0 (17.0) |

*Abbreviations:* ANA, antinuclear antibodies; ACA, anti-centromere antibodies; Anti-RNAPIII, anti-RNA-polymerase III antibodies; mRSS, modified Rodnan skin score; PH, pulmonary hypertension; PAH, pulmonary arterial hypertension; ILD, interstitial lung disease; FVC, forced vital capacity; DLCO, diffusing capacity of the lung for carbon oxide / single breath; ERAs, endothelin receptor antagonists; PDE5i, phosphodiesterase 5 inhibitors; sGC, soluble guanylate cyclase; MMF / MFA, mycophenolate mofetil / mycophenolic acid; CYC, cyclophosphamide; AZA, azathioprine; MTX, methotrexate; TCZ, tocilizumab; RTX, rituximab; GCs, glucocorticoids; JAKi, Janus kinase inhibitors; ARA, angiotensin receptor antagonists; BBs, betablockers; CCBs, calcium channel blockers; ACEi, angiotensin converting enzyme inhibitors.

**Supplementary Table 4. Baseline demographic and clinical characteristics of SSc patients treated in before and after the approval of nintedanib and the positive clinical trials with rituximab and tocilizumab**

| Variable | Overall | After 2021 | Before 2021 |
| --- | --- | --- | --- |
| N | 6583 | 803 | 5780 |
| Male (%) | 1336.0 (20.4) | 189.0 (23.8) | 1147.0 (20.0) |
| Age (mean (SD)) | 55.28 (13.79) | 55.91 (14.10) | 55.19 (13.74) |
| Follow-up (years, mean (SD)) | 4.10 (5.02) | 0.76 (1.05) | 4.56 (5.18) |
| Number of visits (mean (SD)) | 3.67 (3.48) | 1.74 (0.98) | 3.93 (3.62) |
| Disease duration (%) |  |  |  |
| - ≤ 3 years | 3038.0 (49.3) | 391.0 (55.4) | 2647.0 (48.5) |
| - 4-6 years | 971.0 (15.7) | 106.0 (15.0) | 865.0 (15.8) |
| - 7-10 years | 854.0 (13.8) | 67.0 (9.5) | 787.0 (14.4) |
| - ≥ 10 years | 1304.0 (21.1) | 142.0 (20.1) | 1162.0 (21.3) |
| ANA positive (%) | 5669.0 (91.0) | 690.0 (92.5) | 4979.0 (90.8) |
| Anti-Scl70 positive (%) | 1889.0 (32.4) | 246.0 37.0) | 1643.0 (31.8) |
| ACA positive (%) | 2263.0 (39.0) | 294.0 (45.7) | 1969.0 (38.2) |
| Anti-RNAPIII positive (%) | 275.0 (4.8) | 67.0 (11.9) | 208.0 (4.0) |
| Organ involvement |  |  |  |
| Skin involvement (%) | 5041.0 (77.8) | **486.0 (61.1)** | **4555.0 (80.2)** |
| mRSS (mean (SD)) | 8.69 (8.52) | 8.42 (8.13) | 8.73 (8.57) |
| Raynaud phenomenon (%) | 6142.0 (94.7) | 736.0 (92.5) | 5406.0 (95.0) |
| Digital ulcers (%) | 1375.0 30.8) | **210.0 (26.4)** | **1165.0 (31.7)** |
| Calcinosis (%) | 651.0 (14.6) | 120.0 (15.1) | 531.0 (14.4) |
| Telangiectasia (%) | 1482.0 (52.2) | 407.0 (53.4) | 1075.0 (51.7) |
| Pulmonary involvement |  |  |  |
| - PH (%) | 747.0 (11.5) | 81.0 (10.2) | 666.0 (11.7) |
| - PAH (%) | 338.0 (5.2) | 44.0 (5.5) | 294.0 (5.2) |
| - ILD (%) | 2095.0 (32.3) | **213.0 (26.8)** | **1882.0 (33.1)** |
| - - ILD with PH (%) | 410.0 (6.3) | 37.0 (4.6) | 373.0 (6.6) |
| - - ILD without PH (%) | 1731.0 (26.8) | 177.0 (22.2) | 1554.0 (27.4) |
| Baseline FVC % (mean (SD)) | 88.22 (20.00) | 87.15 (19.13) | 88.63 (20.32) |
| Baseline DLCO % (mean (SD)) | 68.32 (21.74) | 65.88 (20.85) | 68.73 (21.86) |
| Cardiac involvement (%) | 732.0 (11.3) | **70.0 (8.8)** | **662.0 (11.7)** |
| Musculoskeletal involvement (%) | 1982.0 (31.4) | **149.0 (18.7)** | **1833.0 (33.2)** |
| Esophageal involvement (%) | 2999.0 (46.3) | **264.0 (33.2)** | **2735.0 (48.2)** |
| Stomach involvement (%) | 783.0 (12.1) | 86.0 (10.8) | 697.0 (12.3) |
| Diarrhea (%) | 1977.0 (31.5) | **113.0 (14.4)** | **1864.0 (33.9)** |
| Malabsorption (%) | 914.0 (22.7) | 173.0 (22.2) | 741.0 (22.8) |
| Renal involvement (%) | 455.0 (7.0) | **28.0 (3.5)** | **427.0 (7.5)** |
| Scleroderma renal crisis (%) | 51.0 (1.2) | 5.0 (0.6) | 46.0 (1.3) |
| Therapy |  |  |  |
| Vasoactive therapy in the last year (%) | 4095.0 (65.2) | 598.0 (76.0) | 3497.0 (63.7) |
| ERA (%) | 652.0 (13.9) | **165.0 (21.6)** | **487.0 (12.4)** |
| PDE5i (%) | 392.0 (8.3) | 72.0 (9.5) | 320.0 (8.1) |
| Prostanoids / prostacyclin receptor agonists (%) | 1488.0 (30.1) | **345.0 (44.6)** | **1143.0 (27.4)** |
| sGC stimulators (%) | 15.0 (0.5) | 5.0 (0.7) | 10.0 (0.5) |
| Nintedanib (%) | 68.0 (1.1) | **61.0 (9.1)** | **7.0 (0.1)** |
| Immunomodulatory therapy last year (%) | 2598.0 (100.0) | 424.0 (100.0) | 2174.0 (100.0) |
| MMF / MFA (%) | 398.0 (7.6) | **117.0 (14.7)** | **281.0 (6.3)** |
| CYC (%) | 400.0 (7.4) | **44.0 (5.5)** | **356.0 (7.7)** |
| AZA (%) | 397.0 (7.4) | **33.0 (4.1)** | **364.0 (7.9)** |
| MTX (%) | 999.0 (18.0) | **169.0 (21.2)** | **830.0 (17.5)** |
| TCZ (%) | 25.0 (0.4) | **24.0 (3.0)** | **1.0 (0.0)** |
| RTX (%) | 29.0 (0.4) | **22.0 (2.8)** | **7.0 (0.1)** |
| Chloroquine / Hydroxychloroquine (%) | 513.0 (9.7) | 116.0 (14.6) | 397.0 (8.8) |
| GCs (%) | 1996.0 (32.2) | **151.0 (19.7)** | **1845.0 (34.0)** |
| Other immunomodulatory therapy (%) | 221.0 (10.5) | 38.0 (6.0) | 183.0 (12.4) |
| JAKi (%) | 4.0 (0.1) | 4.0 (0.5) | 0.0 (0.0) |
| ARA (%) | 19.0 (0.5) | 1.0 (0.2) | 18.0 (0.5) |
| BB (%) | 10.0 (0.2) | 2.0 (0.3) | 8.0 (0.2) |
| CCB (%) | 1837.0 (34.0) | 268.0 (34.7) | 1569.0 (33.8) |
| ACEi (%) | 1281.0 (20.8) | **99.0 (12.9)** | **1182.0 (21.9)** |
| Other vasodilators (%) | 56.0 (2.7) | 12.0 (1.8) | 44.0 (3.1) |
| Malignancy ever (%) | 644.0 (16.4) | 93.0 (12.0) | 551.0 (17.5) |

*Abbreviations:* ANA, antinuclear antibodies; ACA, anti-centromere antibodies; Anti-RNAPIII, anti-RNA-polymerase III antibodies; mRSS, modified Rodnan skin score; PH, pulmonary hypertension; PAH, pulmonary arterial hypertension; ILD, interstitial lung disease; FVC, forced vital capacity; DLCO, diffusing capacity of the lung for carbon oxide / single breath; ERAs, endothelin receptor antagonists; PDE5i, phosphodiesterase 5 inhibitors; sGC, soluble guanylate cyclase; MMF / MFA, mycophenolate mofetil / mycophenolic acid; CYC, cyclophosphamide; AZA, azathioprine; MTX, methotrexate; TCZ, tocilizumab; RTX, rituximab; GCs, glucocorticoids; JAKi, Janus kinase inhibitors; ARA, angiotensin receptor antagonists; BBs, betablockers; CCBs, calcium channel blockers; ACEi, angiotensin converting enzyme inhibitors.
